# Landscape condition influences energetics, reproduction, and stress biomarkers in grizzly bears

**DOI:** 10.1038/s41598-021-91595-4

**Published:** 2021-06-09

**Authors:** Abbey E. Wilson, Dan Wismer, Gordon Stenhouse, Nicholas C. Coops, David M. Janz

**Affiliations:** 1 Department of Veterinary Biomedical Sciences, University of Saskatchewan, 52 Campus Drive, Saskatoon, SK S7N 5B4 Canada; 2 Department of Forest Resource Management, University of British Columbia, 2424 Main Mall, Vancouver, BC V6T 1Z4 Canada; 3 fRI Research, Grizzly Bear Program, 1176 Switzer Drive, Hinton, AB T7V 1V3 Canada; 4 Toxicology Centre, 44 Campus Drive, Saskatoon, SK S7N 5B3 Canada

**Keywords:** Physiology, Ecology

## Abstract

Environmental change has been shown to influence mammalian distribution, habitat use, and behavior; however, few studies have investigated the impact on physiological function. This study aimed to determine the influence of landscape condition on the expression of target proteins related to energetics, reproduction, and stress in grizzly bears. We hypothesized that changes in landscape condition explains protein expression. Skin biopsies were collected from free-ranging grizzly bears in Alberta, Canada from 2013–2019 (n = 86 individuals). We used an information theoretic approach to develop 11 a priori candidate generalized linear mixed models to explain protein expression. We compared models using Akaike Information Criteria (AICc) weights and averaged models with ΔAICc < 2 for each protein. Food resources, represented by increased distance to coal mines and decreased crown closure, positively influenced energetic proteins (adiponectin and alpha-1-acid glycoprotein). Proteins related to reproduction (ceruloplasmin and serpin B5) were positively associated with increased wetland and upland food resources in addition to movement, but negatively associated with increased distance to roads. One stress related protein, complement C3, was positively influenced by increased percent conifer. Given the need to detect emerging threats to wildlife, we suggest the assessment of physiological function will lead to improved monitoring of species in rapidly changing landscapes.

## Introduction

Increased agricultural land use and urban development has resulted in significant habitat losses and ultimately, the endangerment of several species across Canada^1^. In the province of Alberta, grizzly bear (*Ursus arctos*) populations reside in areas with an increasing demand for natural resources, agriculture, and recreation, all of which lead to increasing fragmentation and loss of habitat^2^. This species was listed as threatened by the province in 2010^3^, which prompted extensive research on the conservation strategies needed to inform policy makers and management activities in the region^4–6^. These strategies included the expansion of our understanding of wildlife health in order to identify, measure, and mitigate potential negative effects of such environmental change on wildlife populations^7^. The concept of wildlife health has been poorly defined and is traditionally thought of as merely the absence of disease^8,9^. However, Stephen (2014) suggests that health should be measured by the characteristics of the individual and their ecosystem that may impact resilience, especially when integrating wildlife health with natural resource and ecosystem conservation^9^. Therefore, there is an increasing need to develop determinants of individual animal health to address the cumulative effects of social, environmental, and biological characteristics of animals in order to measure and monitor wildlife health at the population level^10^. In response to this growing demand, federal, provincial, and territorial Ministers responsible for biodiversity and conservation in Canada approved a new Pan-Canadian Approach to Wildlife Health in 2018, which stresses the need to detect emerging threats to wildlife and provide early warning of potential population decline^11^. It has been recognized that rapidly changing environments can influence the immune system and ultimately affect reproductive success in wildlife^7^; however, there is a substantial knowledge gap regarding the impact of environmental disturbance on animal health and the physiological mechanisms by which individuals respond to such change.

Recently, the pace-of-life syndrome has been proposed as a new approach to understanding animal responses to environmental conditions by suggesting that life history and physiological traits have evolved together in response to the environment^12^. The pace-of-life syndrome hypothesis predicts that individual differences in behavior are related to morphological, physiological, and life-history traits along a slow to fast pace-of-life scale and these differences can be assessed within populations, between populations, and between species^13,14^. Life history theory has traditionally included variables related to biological functions such as maintenance, growth, and reproduction, that influence survival and reproductive success, all of which are regulated by physiological mechanisms^12^. Given the relationship between life history characteristics and physiological mechanisms (hormonal, metabolic, and immunological), both are thought to be influenced by ecological conditions, which may favor certain strategies and/or physiological traits. For example, climate has been shown to influence the basal metabolic rate of closely related small mammals along a slow-fast metabolic continuum, where the fast end of the continuum (high metabolic rate) was associated with high latitudes and mean productivity, and the slow end (low metabolic rate) was associated with low productivity zones^15^. Therefore, individuals residing in environments with limited resources will likely have lower metabolic rates and a slower pace-of-life (later age of maturity, fewer offspring, and higher life expectancy) compared to those living in areas with high quality and abundant resources^12,13^. Large mammals, such as grizzly bears, tend to exhibit a slow pace-of-life, with a slower growth rate, delayed age of first reproduction, and large interbirth interval^16^. These life history characteristics have been shown to be highly variable across the species’ geographic range and are influenced by food availability^17^. Understanding relationships between physiological function and changing environments may provide further insight into life-history variation and adaptive responses in wildlife.

The use of contemporary molecular tools to determine physiological function may provide novel approaches to detect parameters that indicate declining health status in a population. These methods offer a means to understand the physiological mechanisms that influence health in this species, thereby aiding scientists and managers with the identification of conservation strategies prior to critical population thresholds, as declining health may influence population performance (e.g., number of cubs produced). Such tools include liquid chromatography coupled with mass spectrometry, which has been applied to the study of proteins (proteomics) over the last decade^18,19^. Proteins are functional molecules generated by the information expressed in the genome and manifested at the message level (mRNA)^19^. However, it is difficult to predict the degree of change in the active mature protein when measuring mRNA (transcriptomics), as protein levels depend on the levels of the messages as well as translational controls and regulated degradation^20^. Additionally, proteins respond to stimuli and help to regulate biological function, thus, it is thought that measuring the expression of proteins may provide the most relevant characterization of a biological system^19^. In particular, liquid chromatography and multiple reaction monitoring mass spectrometry (LC-MRM/MS) assays have been developed to detect and quantify specific target proteins for hypothesis-driven experiments. Compared to traditional proteomic technologies such as western blotting, enzyme-linked immunosorbent assays (ELISA), or even untargeted or global proteomic approaches, LC-MRM/MS assays provide higher sensitivity (detection limits at femtomole amounts), selectivity (quantification across 4–5 orders of linear dynamic range), and reproducibility (< 13% coefficient of variation when compared to commercially available immunoassays)^21,22^. Sources of irreproducibility using a targeted approach have been identified when comparing across multiple laboratories, which include unpredictability of the LC system and saturation of the MS detector^23^. However, using internal standards across sample batches and laboratories can serve as a means of quality control for instrument variability, while measuring pooled samples can account for sample variability across replicates (see Wilson et al., 2020 for information on target proteins in this study)^23–25^. There has been increased interest and success in the identification and use of protein biomarkers to predict clinical outcomes in humans^26^, as specific proteins can serve as indicators of physiological or pathological states and thus provide information for the early detection of poor health and/or disease^27^.

Our previous study identified and quantified 19 target proteins related to energetics, reproduction, and stress in the skin of free-ranging grizzly bears^28^. Skin has been shown to have a unique process for responding to stress and other stimuli, as skin cells produce elements of the hypothalamic–pituitary–adrenal (HPA) and hypothalamic–pituitary–thyroid (HPT) axes, both of which regulate metabolism and stress^29,30^. Proteins expressed in skin may serve as potential biomarkers of health due to their ability to regulate homeostasis, respond to signals, and maintain physiological function^30,31^. For example, proteins related to energetics, such as adiponectin, clusterin and apolipoprotein are involved in the regulation of glucose, transporting nutrients, and clearing cellular debris and can serve as biomarkers for metabolic and cardiovascular disease^32–34^. Proteins related to reproduction, such as ceruloplasmin, fetuin-B, and prostaglandin (PG) F synthase 1 transport nutrients for pregnancy, facilitate fertilization, and initiate parturition. These proteins can be potential indicators for pregnancy and fertility^35–37^. Lastly, proteins involved in the stress response including corticosteroid-binding globulin (CBG), endoplasmin, and annexin are involved in transporting stress hormones, maintaining protein function, and reducing inflammation. Changes in the expression of these proteins may suggest acute and chronic stress, pathogenesis, and inflammatory disease^38–40^. However, the expression of several proteins was highly correlated; therefore, in an attempt to not measure the same signal unnecessarily, highly correlated proteins were removed so that at least one representative of each category was present as well as those that may be suitable biomarkers based on previous grizzly bear studies and according to specific management questions (Table 1). For example, Transthyretin is a biomarker for protein-calorie malnutrition, which may be a useful biomarker of body condition and thus provide information on reproductive ability. While proteins were separated into functional categories for the sake of the current study, it is common for a suite of proteins to be expressed in order to maintain biological function and homeostasis^41^. Several of the proteins related to reproduction in the present study are also involved in metabolic regulation and the immune response^42,43^. Given these established relationships, we aim to address the need for better understanding the relationships between of environmental condition and wildlife health by investigating the influence of landscape disturbance on protein expression.

**Table 1:** The influence of landscape condition was assessed to predict the expression of target proteins quantified in grizzly bear skin related to energetics, reproduction, and stress. Table modified from Wilson et al., 2020.

Category	Protein	Biomarker	Reference
Energetics	Adiponectin	Metabolic disease	^32^
	Apolipoprotein B-100	Cardiovascular disease	^34^
	Alpha-1-acid glycoprotein	Inflammation and liver disease	^44^
	Transthyretin	Protein-calorie malnutrition	^45^
Reproduction	Ceruloplasmin	Pregnancy	^35,46^
	Fetuin-B	Female fertility	^36,47^
	Serpin B5 (Maspin)	Pregnancy-associated disorders	^48,49^
Stress	Superoxide dismutase	Inflammatory and infectious disease	^50^
	Corticosteroid-binding globulin	Acute and chronic inflammation	^38^
	Complement C3	Impaired immune response	^43,51^

The goal of this study was to determine how landscape condition may be influencing physiological function in grizzly bears. As human use and alteration of the landscape continues to increase, better links between landscape resources, resource use and extraction, and wildlife health need to be developed, allowing resource managers to set realistic science-based goals and quantifiable disturbance thresholds to support and guide sustainable land management decisions. The objective of this study was to determine the influence of landscape condition on the expression of target proteins related to energetics, reproduction, and stress in grizzly bears. Given that landscape disturbance can alter food availability, increase mortality risk, and influence individual behavior, we hypothesized that changes in landscape condition explain differences in protein expression.

We used an information theoretic approach to develop 11 a priori candidate generalized linear mixed models with corresponding hypotheses that described plausible relationships between protein expression and landscape variables based on the literature to explain protein expression^52–55^. We used generalized linear mixed model analysis to develop the candidate models with watershed unit and elevation combination as a random effect to account for any spatial dependency, compared them by Akaike Information Criteria (AICc) weights, and averaged the minimum adequate models with ΔAICc < 2 for the expression of each protein (Table 2).

**Table 2:** A priori candidate generalized linear mixed models to explain protein expression in skin collected from grizzly bears in Alberta, Canada.

Model	Expression	Hypothesis	Reference
M1	Distance to roads + (1|watershed unit)	Roads influence protein expression by creating attractive sink habitat due to increased foods and higher probability of mortality	^52,56^
M2	Distance to rail/power lines + (1|watershed unit)	Railways and powerlines influence protein expression by providing food resources, edge habitat, and areas largely away from motorized traffic	^52^
M3	Cutblock age + (1|watershed unit)	Younger forest stands will have higher food resources that impact protein expression	^52,56^
M4	Distance to coal mines + (1|watershed unit)	Coal mines have low vegetation but high ungulate density as food resources, and predictable levels of human use that affect protein expression	^57^
M5	Protected area + (1|watershed unit)	Protected areas are comprised of mostly old forest (less food availability) with relatively low levels of human use that influences protein expression	^53,58^
M6	Percent conifer + (1|watershed unit)	The species composition of trees in the area will dictate behavior and consequently protein expression	^59,60^
M7	Crown closure + (1|watershed unit)	Poor food resources in forests with increased crown closure will influence protein expression	^61^
M8	Upland herbaceous area + Wetland herbaceous area + (1|watershed unit)	Herbaceous resources around water and shrubs provide food resources that will affect protein expression	^58,62^
M9	Mean movement + (1|watershed unit)	Increased movement from searching for food and mates influences protein expression	^55^
M10	Reproductive class + Age class + (1|watershed unit)	Changes in physiology due to sexual maturity or having offspring influences protein expression	^28^
M11	Null	None of the covariates affect protein expression	

We predicted that variables related to anthropogenic disturbance would be associated with increased expression of proteins related to stress, as previous studies have demonstrated a relationship between human use on the landscape and long term stress in grizzly bears^52,63^. Increased reproductive success has been associated with increased food resources in several species and in particular, primary habitat for grizzly bear reproduction has been shown to have specific land cover attributes^58,62^. Therefore, we predicted that variables related to food resources would be associated with the expression of proteins related to reproduction. Lastly, we predicted that variables related to food resources and terrain conditions would influence the expression of proteins related to energetics. Such proteins are involved in the regulation of metabolism and metabolic demands, which is influenced by activity and movement^55,64–66^. To our knowledge, this is the first study to assess the impact of landscape condition on the expression of proteins related to energetics, reproduction, and stress in any free-ranging mammal. We aim to provide a novel method by which scientists and managers can further assess changing environmental conditions and how these may impact physiological function in individuals, thus providing new approaches to monitor species-at-risk.

## Results

Between 2013 and 2019, 103 skin samples were collected from free-ranging GPS-collared grizzly bears in Alberta, Canada. However, three individuals had < 50 GPS points within a season and seven individuals had been translocated > 65 km and thus were excluded from the analysis. Furthermore, two samples had been collected from seven individuals over multiple years (n = 6 over 2 years and n = 1 over 3 years); however, only the sample with the greatest skin mass and total protein concentration was used to determine the influence of landscape condition on protein expression. This resulted in a full sample size of 86 samples collected from 86 individuals for the overall analysis. The subset of individuals with multiple samples collected was used to investigate differences in protein expression across years and the subset of individuals that were translocated > 65 km were used to determine if protein expression differed between translocated (n = 7) and resident (n = 7) individuals in the same age-sex class, season, and bear management area.

**Figure 1:**
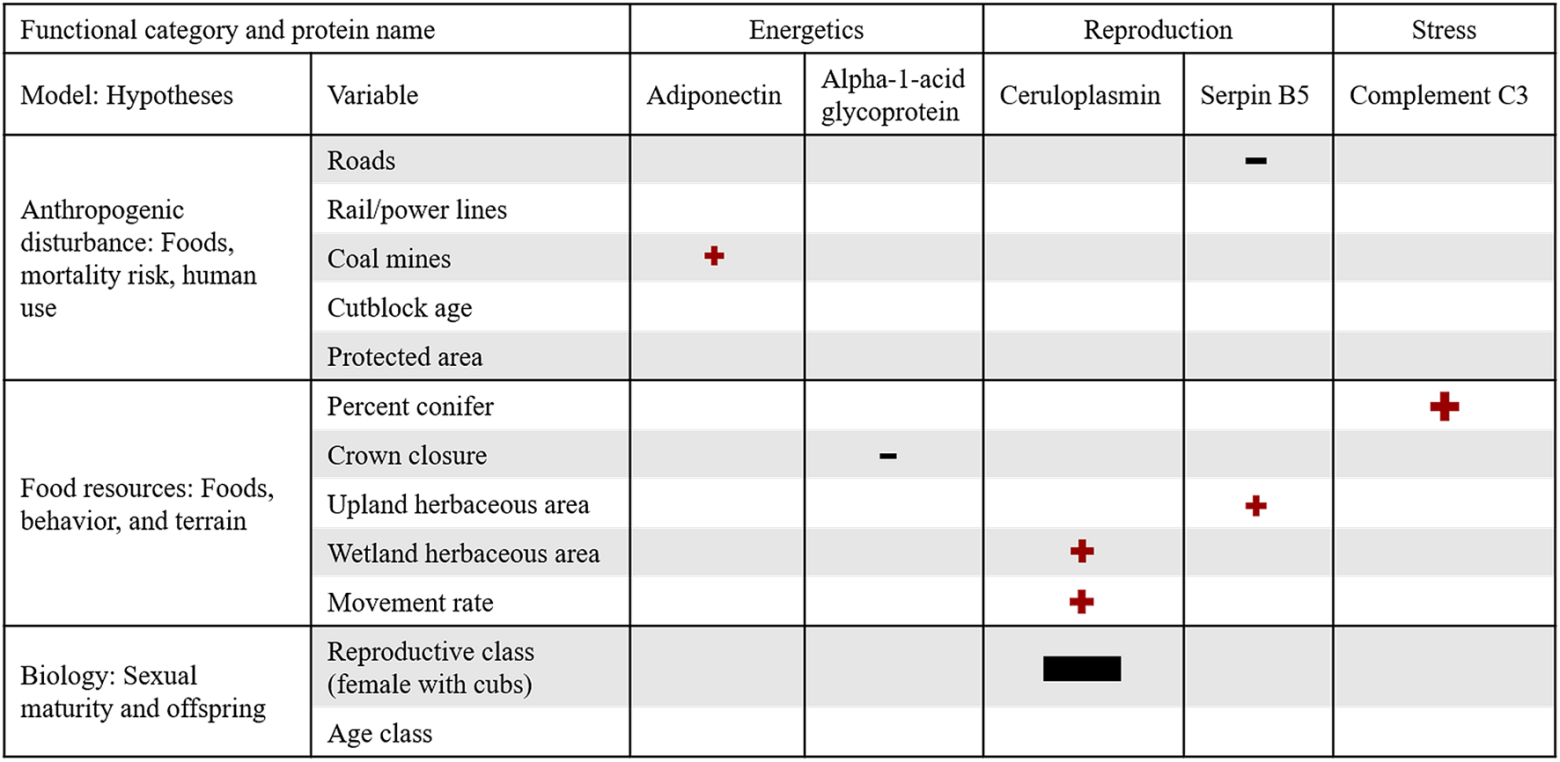
Standardized coefficients represented by symbols for the significant (95% confidence interval does not include zero) parameters in the minimum adequate models (model selection by AICc < 2) with corresponding hypotheses for predicting protein expression in skin samples collected from grizzly bears across Alberta, Canada. The shape and color of each symbol indicates the relationship (red positive sign = direct and grey negative sign = inverse) and the size of each symbol indicates the magnitude of the coefficient estimate.

Variables related to food resources with corresponding hypotheses associated with foods, individual behavior, and terrain influenced the expression of the greatest number of proteins, specifically those related to energetics (alpha-1-acid glycoprotein), reproduction (ceruloplasmin and serpin B5), and stress (complement C3; Fig. 1). Variables related to anthropogenic disturbance with hypotheses associated with foods, mortality risk and human use influenced the expression of proteins related to metabolism (adiponectin) and reproduction (serpin B5), while biology influenced one protein related to reproduction (ceruloplasmin; Fig. 1). In the subset of individuals where samples were collected in 2 years, protein expression between the two time points was not significantly (P > 0.05) different for any protein. In the subset of individuals where samples were collected from translocated and resident bears, the expression of superoxide dismutase was significantly (P < 0.05) elevated in resident bears compared to translocated bears (see Supplementary Figure S1 online).

### Proteins related to energetics

**Figure 2:**
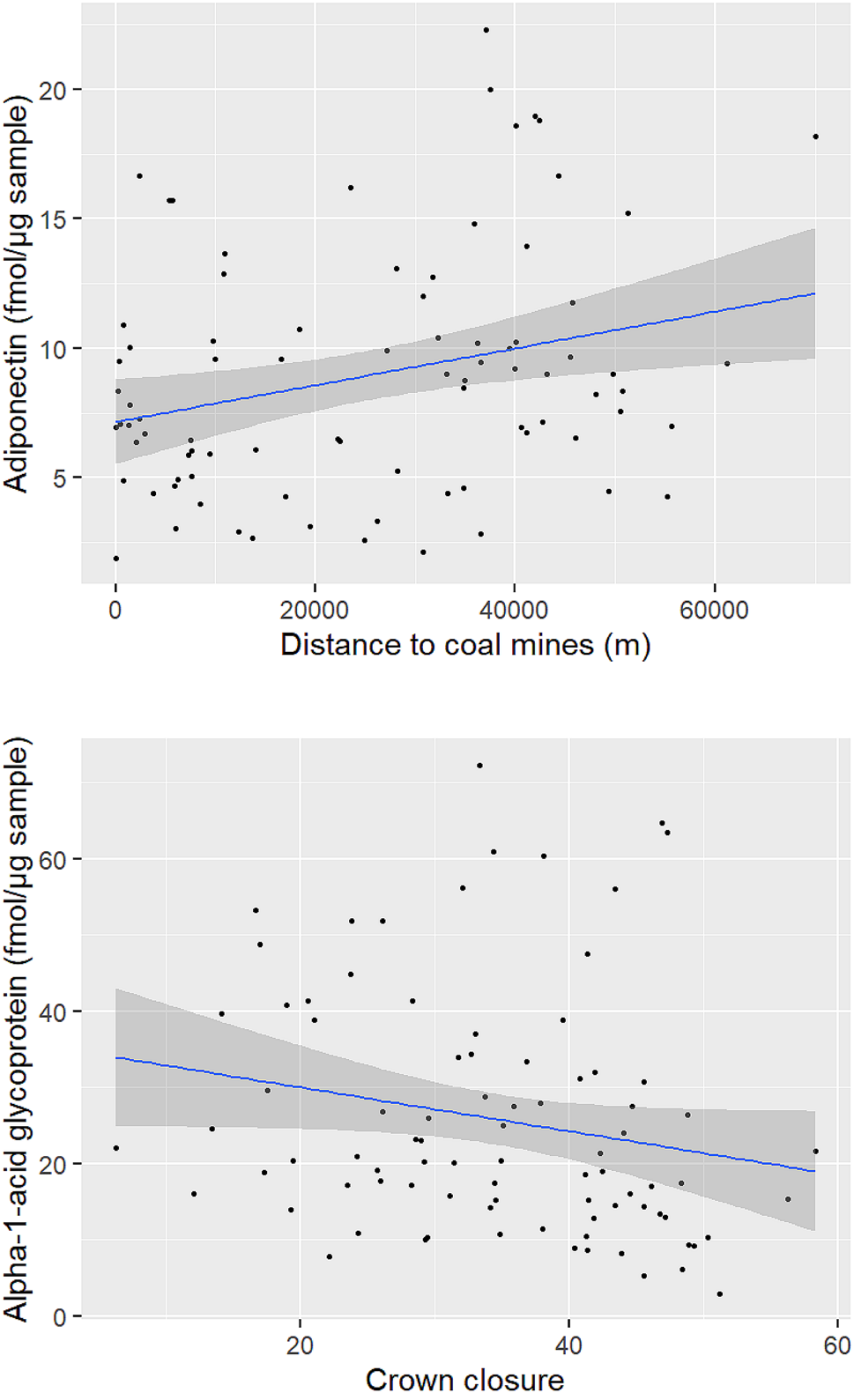
Estimated expression (solid blue line) of adiponectin (top panel) and alpha-1-acid glycoprotein (bottom panel) related to the distance to coal mines and crown closure, respectively. Grey shade is 95% confidence band and closed symbols are the raw data of protein expression. Figure was created using R statistical software version 4.0.3 and R studio version 1.3.1093 (https://www.R-project.org/).

In the set of a priori models, ΔAICc < 2 was achieved with M4 for adiponectin and M7 and M6 for alpha-1-acid glycoprotein (see Supplementary Table S1 online). For the remaining proteins related to energetics, our predictor variables did not explain the observed variation in the data significantly better than the null model. As a result, we selected the model with distance to coal mines as the adequate model to explain the expression of adiponectin, while the models containing percent conifer and crown closure were averaged to explain the expression of alpha-1-acid glycoprotein. In the selected model for adiponectin, we found that as bears moved further away from coal mines, adiponectin increased (Table 3). For alpha-1-acid glycoprotein, as crown closure decreased and thus the forest canopy opened, the expression of alpha-1-acid glycoprotein increased (Table 3). The expression of adiponectin increased over the standardized range of distance to coal mines and alpha-1-acid glycoprotein decreased over the consistent range of standardized crown closure (Fig. 2).

**Table 3:** Estimate (β), standard error (SE) and 95% confidence interval (CI) of the explanation variables in models with ΔAICc < 2 for the expression of adiponectin and alpha-1-acid glycoprotein in skin samples collected from grizzly bears across Alberta, Canada. Variables with 95% CI that do not cross zero are shown in bold text.

Protein	Variable	β	SE	95% CI	
Adiponectin	Intercept	2.181	0.075	2.034	2.328
	**Distance to coal mines**	**0.157**	**0.054**	**0.051**	**0.263**
Alpha-1-acid glycoprotein	Intercept	3.244	0.088	3.069	3.418
	**Crown closure**	− **0.143**	**0.070**	− **0.283**	− **0.003**
	Percent conifer	0.121	0.072	− 0.022	0.264
	Upland herbaceous resources	0.110	0.065	− 0.020	0.240
	Wetland herbaceous resources	0.097	0.064	− 0.029	0.224

### Proteins related to reproduction

**Figure 3:**
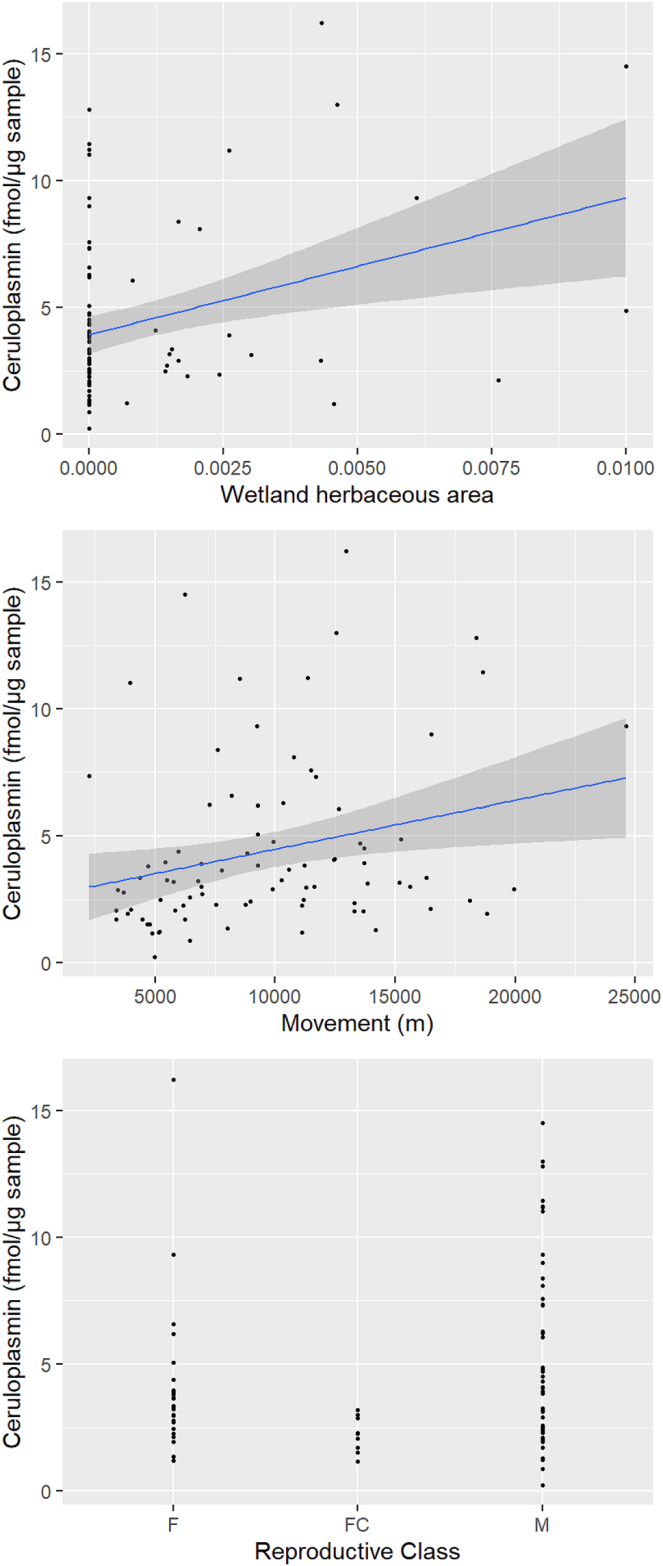
Estimated expression (solid blue line) of ceruloplasmin related to the wetland herbaceous area, movement, and reproductive class (*F* female, *FC* female with cubs, *M* male). Grey shade is 95% confidence band and closed symbols are the raw data of protein expression. Figure was created using R statistical software version 4.0.3 and R studio version 1.3.1093 (https://www.R-project.org/).

**Figure 4:**
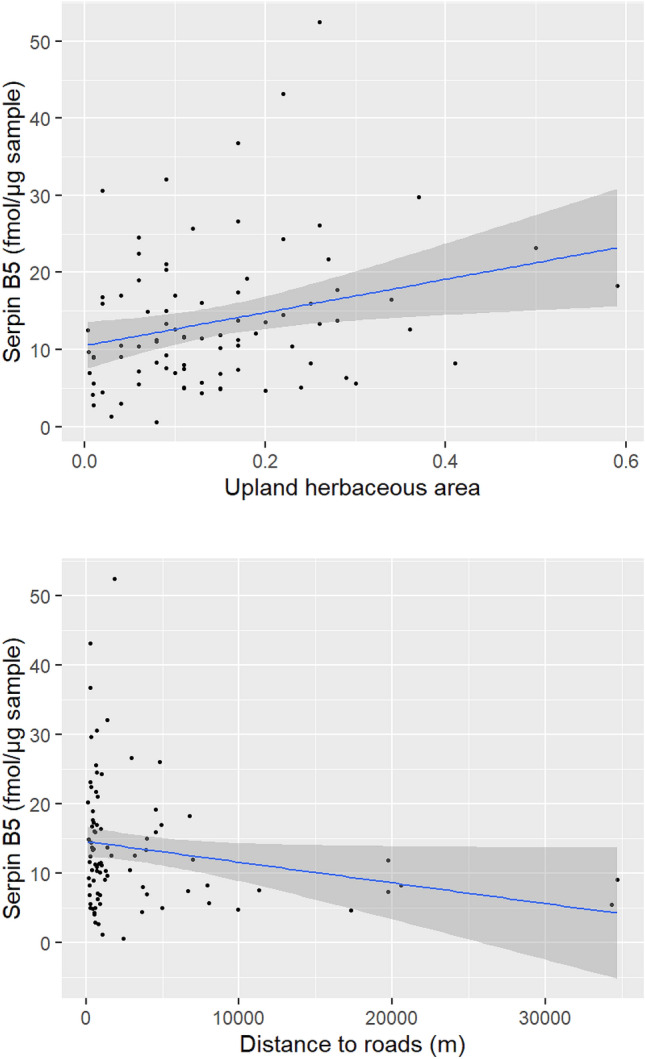
Estimated expression (solid blue line) of serpin B5 related to the distance to upland herbaceous area (top panel) and distance to roads (bottom panel). Grey shade is 95% confidence band and closed symbols are the raw data of protein expression. Figure was created using R statistical software version 4.0.3 and R studio version 1.3.1093 (https://www.R-project.org/).

In the set of a priori models, ΔAICc < 2 was achieved with M10, M8, and M9 to explain the expression of ceruloplasmin and M8 and M1 to explain serpin B5 (see Supplementary Table S1 online). For the remaining proteins related to reproduction, our predictor variables did not explain the observed variation in the data significantly better than the null model. The models containing reproductive class, wetland and upland herbaceous areas, and daily movement were averaged to explain the expression of ceruloplasmin. Similarly, models containing wetland and upland herbaceous areas and distance to roads were averaged to explain the expression of serpin B5. In the averaged models for ceruloplasmin, we found that bears that spent more time in areas with increased wetland herbaceous resources and bears that had a higher movement rate showed an increase in ceruloplasmin (Table 4). Additionally, females with cubs had lower levels of ceruloplasmin when compared to males and solitary females (Table 4). In the averaged models for serpin B5, we found that bears that spent more time in areas with increased upland herbaceous resources, typically areas with shrubs and food resources near water, and that were closer to roads demonstrated an increase in serpin B5 (Table 4). The expression of ceruloplasmin increased over the standardized range of wetland herbaceous areas and movement, but decreased in females with cubs (Fig. 3). The expression of serpin B5 increased over the standardized range of upland herbaceous areas, but decreased over the range of distance to roads (Fig. 4).

**Table 4:** Estimate (β), standard error (SE) and 95% confidence interval (CI) of the explanation variables in models with ΔAICc < 2 for the expression of ceruloplasmin and serpin B5 in skin samples collected from grizzly bears across Alberta, Canada. Variables with 95% CI that do not cross zero are shown in bold text.

Protein	Variable	β	SE	95% CI	
Ceruloplasmin	Intercept	1.444	0.106	1.233	1.654
	**Reproductive class (female with cubs)**	− **0.649**	**0.245**	− **1.136**	− **0.161**
	Reproductive class (male)	0.244	0.154	− 0.062	0.551
	Age class (subadult)	− 0.101	0.155	− 0.410	0.208
	Upland herbaceous resources	− 0.101	0.071	− 0.242	0.039
	**Wetland herbaceous area**	**0.188**	**0.073**	**0.043**	**0.334**
	**Daily movement**	**0.196**	**0.073**	**0.052**	**0.341**
Serpin B5	Intercept	2.591	0.087	2.417	2.764
	**Upland herbaceous area**	**0.167**	**0.071**	**0.026**	**0.307**
	Wetland herbaceous resources	− 0.109	0.073	− 0.255	0.036
	**Distance to roads**	− **0.169**	**0.068**	− **0.305**	− **0.034**

### Proteins related to stress

**Figure 5:**
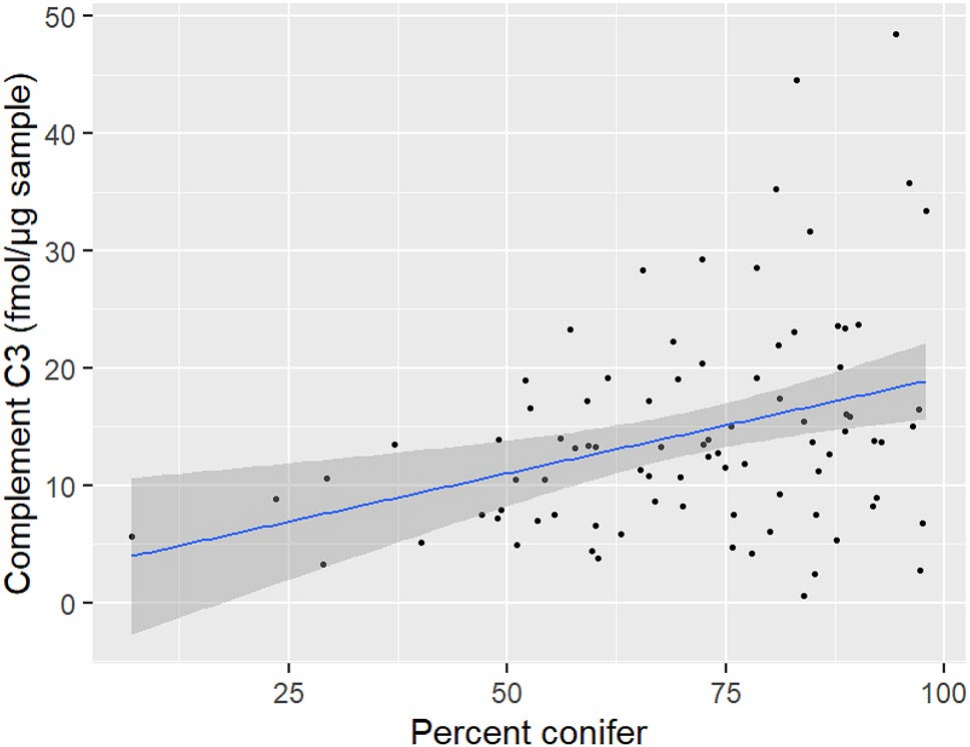
Estimated expression (solid blue line) of complement C3 related to percent conifer. Grey shade is 95% confidence band and closed symbols are the raw data of protein expression. Figure was created using R statistical software version 4.0.3 and R studio version 1.3.1093 (https://www.R-project.org/).

In the set of a priori models, ΔAICc < 2 was achieved with M6 to explain the expression of complement C3 (see Supplementary Table S1online). For the remaining proteins related to stress, our predictor variables did not explain the observed variation in the data significantly better than the null model. As a result, we selected the model with percent conifer as the adequate model to explain the expression of complement C3. In the selected model for complement C3, we found that the expression of complement C3 increased as bears spent time in areas with increased percent conifer that commonly represents pure stand forests with limited food resources (Table 5). The expression of complement C3 increased over the standardized range of percent conifer (Fig. 5).

**Table 5:** Estimate (β), standard error (SE) and 95% confidence interval (CI) of the explanation variables in models with ΔAICc < 2 for the expression of complement C3 in skin samples collected from grizzly bears across Alberta, Canada. Variables with 95% CI that do not cross zero are shown in bold text.

Protein	Parameter	β	SE	95% CI	
Complement C3	Intercept	2.647	0.064	2.522	2.772
	**Percent conifer**	**0.240**	**0.065**	**0.113**	**0.368**

## Discussion

We determined the effects of landscape condition on the expression of proteins in skin related to energetics, reproduction, and stress in grizzly bears. Major findings from this study were that first, food resources, represented by increased distance to coal mines and decreased crown closure, positively influenced proteins related to energetics (adiponectin and alpha-1-acid glycoprotein). Second, proteins related to reproduction (ceruloplasmin and serpin B5) were positively associated with increased wetland and upland food resources in addition to movement, but negatively associated with increased distance to roads. Additionally, females with cubs had lower levels of ceruloplasmin when compared to males and solitary females. Third, one stress related protein, complement C3, was positively influenced by increased percent conifer.

In humans, adiponectin is used as a biomarker of metabolic disease, with decreased levels associated with increased obesity and metabolic disease^32^. Specifically, adiponectin regulates insulin resistance and fatty acid oxidation, which promote the breakdown of glucose and fatty acids consumed in the diet^67^. However, previous studies with captive grizzly bears showed that adiponectin serum concentrations were positively correlated with a high saturated fatty acid diet and percent body fat, and increased gradually throughout the active season and peak in mid-October, prior to hibernation^68,69^. Thus, adiponectin may serve a different physiologic process in bears compared to humans and appears to be correlated with high fatty diet. Coal mines within our study area tend to have less vegetation compared to other areas on the landscape, but are high in ungulate density as food resources for grizzly bears^57^. Our results suggest that bears may find richer food resources, represented by an increase in the expression of adiponectin, as they moved further away from coal mines, perhaps due to an increase in the complexity of the landscape that may facilitate the growth of broader food resources (e.g., berry patches) rather than ungulates alone. In combination with previous studies, the expression of adiponectin may provide insights on body condition and diet of individual grizzly bears. Alpha-1-acid glycoprotein, also called orosomucoid, is influenced by metabolic signals such as insulin, high glucose, and free fatty acid, and has been used as a biomarker of inflammation, liver disease, and heart failure in humans^44^. Previous studies suggest that alpha-1-acid glycoprotein can protect tissues from inflammation and metabolic dysfunction by integrating inflammatory and metabolic signals to regulate immune responses^70^. For example, in Iberian ibex (*Capra pyrenaica*), serum concentrations of alpha-1-acid glycoprotein not only increased in animals that were infected with sarcoptic mange, but also increased in accordance with the severity of sarcoptic mange, suggesting it’s utility as a biomarker for potential organ failure^71^. Our data suggest that the expression of alpha-1-acid glycoprotein decreased as crown closure, a common variable used to estimate the forest understory vegetation abundance^52^, increased. An increase in crown closure is typically associated with poor food resources as the forest canopy does not allow for light to reach the forest understory and thereby decreases the vegetation available for grizzly bears^61^. Decreased levels of alpha-1-acid glycoprotein expression in bears that were in areas with poor food resources may be a result of the role that this protein plays in protecting against metabolic dysfunction, as low levels have been shown to be related to poor nutritional status in humans with chronic heart failure^72^. During hibernation, grizzly bears employ a series of metabolic adaptations, such as metabolism regulation, activation of signaling pathways to protect against muscle atrophy, and lower expression of genes associated with insulin signaling, to limit the negative effects of malnutrition and muscle disuse^73–75^. Similar to adiponectin, the expression of alpha-1-acid glycoprotein may have different physiological effects in animals that enter a period of hyperphasia compared to other non-hibernating mammals.

In our study area, wetland and upland herbaceous areas consisted of open and treed wetlands that could occur in valley bottoms as well as high elevation depressions, which tend to be selected by grizzly bears, likely due to the increased food resources and water available in such areas^59,76^. Additionally, potential habitat quality for grizzly bears has been reported to be highest in low elevation mountain valleys, where fruiting resources such as buffaloberries (*Shepherdia canadensis*) and rooting resources such as sweet vetch (*Hedysarum* spp.), tend to occur^77^. Thus, it is not surprising that the expression of reproductive proteins (ceruloplasmin and serpin B5) would be higher in areas with high food resources. Ceruloplasmin evolved to have multifunctional properties that also involve transferring electrons to oxygen in some metabolic pathways of vertebrates^78^, which supports our finding of increased expression of ceruloplasmin when movement rate increases. Previous studies show that bears have an energetic cost for locomotion and this cost doubles as their speed increases^79^. This energetic cost intensifies when high metabolic rates needed for foraging are linked with a decreased or unbalanced intake of macronutrients, as described in polar bears^55^. Because habitat type has been shown to influence grizzly bear activity patterns, with most foraging behavior occurring in recently disturbed forest with herbaceous food resources and open-canopy forests^59^, ceruloplasmin may be elevated when foraging (moving more) for food resources. Additionally, ceruloplasmin has been used as a biomarker of pregnancy in several mammals^35^. For example, it was found to be elevated in the urine of giant pandas (*Ailuropoda melanoleuca*) during pregnancy^46^. This is likely due to the relationship between ceruloplasmin and estrogen and progesterone, as these hormones have well established enhancing effects on the expression of ceruloplasmin^35^. This may explain why ceruloplasmin was found to be lower in females with cubs compared to solitary females and males, as females with cubs are likely not experiencing an estrous cycle or pregnancy, and thus have lower concentrations of estrogen and progesterone. Similar to ceruloplasmin, serpin B5, or maspin, has been shown to be related to pregnancy in humans. In a previous study, the expression of serpin B5 was elevated in preeclamptic pregnancies compared to normal pregnancies and the authors suggest that this protein may be used to detect pregnancy associated disorders^49^. There were no known cases of pregnancy associated disorders in the current study; therefore, the current study cannot assess the utility of serpin B5 as a biomarker of pregnancy complications; however, it may provide insights on reproductive ability in the future. Given the role of serpin B5, we expected model selection to result in support for the biology model (reproductive and age class), yet the expression of serpin B5 was negatively associated with increased distance to roads, suggesting that as individuals moved closer to roads, the expression of serpin B5 also increased. In avian species, forest fragmentation and changes to forest composition (i.e., roads) have been shown to influence the quality and distribution of high-quality habitat, which in turn has been shown to negatively impact reproductive success^80^. However, poor reproduction has not been observed in this population and therefore, protein expression levels related to reduced reproductive output are not yet known. Solitary females and females with cubs have been shown to spend more time in close proximity to unpaved roads, especially those that are near creeks and in open areas with high vegetative cover, representing high quality habitat for grizzly bears^56,81^. Conversely, other studies have shown solitary females avoid public roads^57^, while males tend to avoid roads altogether^57,82^. Elevated levels of stress have been reported to be correlated with reduced reproductive output in humans^83^ and in this case, elevated levels of proteins related to pregnancy disorders may be influenced by the mortality risk associated with the increase in roads and other access features that are built to support natural resource extraction activities^6,84,85^. While the physiological mechanisms for the expression of serpin B5 are not fully understood for this species, measuring this protein may be a potential tool to help determine reproductive issues in females, such as why some adult females in our population have never been seen with cubs.

Both high and low levels of complement C3 have potential to be biomarkers for different aspects of immunity in wildlife. In a previous study, complement C3 was shown to increase in rainbow trout in response to an acute stressor, thereby increasing the efficacy of their natural immune system^51^. Therefore, increased expression of complement C3 may be indicative of a stress response; however, complement C3 deficiency has been associated with increased susceptibility to bacterial infections in other studies^43^, suggesting that if individuals do not have enough complement C3, then their immune response may be impaired. The expression of complement C3 was positively influenced by increased percent conifer, indicating that complement C3 increased as the species composition of trees in the area became more uniform. Open conifer stands provide high quality habitat for grizzly bears by facilitating the growth of rooting resources, while closed conifer stands have been shown to enable the growth of black huckleberry (*Gaylussacia baccata*), both of which are important food resources for grizzly bears^77^. However, pure conifer stands compared to mixed wood stands tend to have fewer food resources for grizzly bears, but are still used for bedding and resting behaviors^77^. In this case, an increase in complement C3 may be indicative of an increased stress response due to low food resources in pure conifer stands.

Broad relationships between landscape condition and the expression of proteins related to energetics, reproduction, and stress were determined for grizzly bears. While landscape variables were chosen based on biologically and ecologically plausible relationships with indicators of bear health, different variables extracted at various spatial scales may further elucidate these relationships. Furthermore, to distinguish the effects of landscape change on protein expression, several years of landscape data may be needed, as we did not detect any influence of landscape change on protein expression when samples were collected across 2 years, likely because the level of landscape change in 1 year is relatively low. However, the expression of superoxide dismutase, a biomarker for stress and inflammation^50^, was elevated in samples collected from a subset of resident bears compared to translocated bears. This may suggest that translocated bears were less stressed before transport, perhaps due to foraging on higher quality food resources (cows, pigs, grains etc.) along with other factors, compared to resident bears. Further investigation is required, but measuring protein expression in translocated and resident bears may provide useful insights into how translocation efforts are impacting physiological function as well as the underlying physiological conditions that may be driving conflict behavior.

### Application for monitoring wildlife

The practical application of this method requires the (1) collection of skin samples from free-ranging animals, (2) development and validation of a LC-MRM/MS assay, and (3) analysis of highly complex physiological and landscape data. Skin samples can be collected from individuals that are live-captured, harvested, or free-ranging with a remote biopsy dart, potentially during other ongoing studies^28,86^. Upfront costs for the development and validation of a proteomic assay are expensive; however, once the assay has been developed, the sample analysis costs are moderate. These costs can be reduced by focusing on key proteins that may serve as biomarkers of physiological function and potentially forecast future population decline, such as those related to pregnancy and pregnancy associated disorders (ceruloplasmin and serpin B5), metabolic disease (adiponectin and alpha-1-acid glycoprotein), and stress/immune function (complement C3). The analysis of highly complex data will need to be conducted by a collaborative team with knowledge in gathering and measuring landscape condition data, physiological data, and statistical analysis. While the development of this technique is in the early stages, we suggest the use of this method to create a “proteomic health assay” that can be used to monitor physiological responses of individuals to landscape condition. This initial study provides a method by which to monitor physiological function at the population level in response to landscape condition as well as important baseline physiological information relative to current landscape features. Further research is needed to determine how or if the expression of particular proteins is indicative of specific population performance parameters (e.g., number of cubs produced) that may provide an early warning of declining health status in populations. We suggest that scientists use these methods to focus on linking key proteins and/or groups of proteins that may serve as biomarkers of population increases or declines with quantifiable population health measures, such as reproduction and survival.

## Methods

### Study area

**Figure 6:**
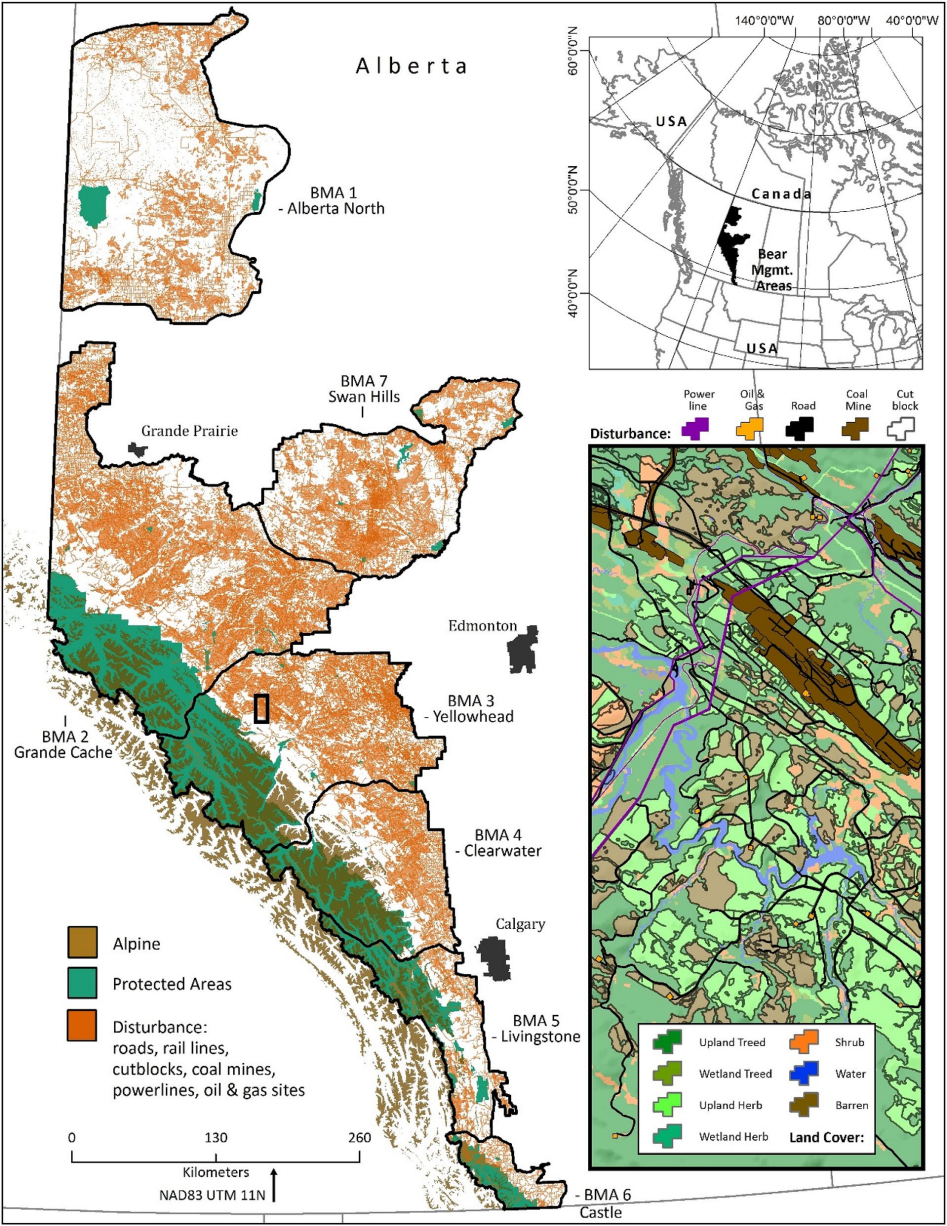
Study area location in Alberta, Canada. Skin samples were collected from free-ranging grizzly bears from 2013–2019 in Clearwater, Yellowhead, and Grande Cache bear management areas (BMA). On main layout, disturbance from ABMI HF118 (http://www.abmi.ca) was grouped and mapped. Alpine and protected parks boundaries were added. On subset map, land cover^91^ was mapped, and ABM HFI disturbance features were added. ArcGIS Desktop 10.3.1 software was used (https://desktop.arcgis.com/en/).

We conducted this study with free-ranging grizzly bears residing in west-central Alberta, Canada (Fig. 6). The study area includes parks and protected areas with little landscape change in the mountainous (3500 m) region to the west as well as areas that are subject to anthropogenic resource use, including forestry, oil and gas exploration, and mining in the foothills to the east^2,87^. Previous studies have documented the forest conditions and grizzly bear food resources present in this study area^52,77,88,89^. Grizzly bears were captured and fitted with a GPS collar by the fRI Research Grizzly Bear Program (FRIGBP, Hinton, AB, Canada) following procedures described elsewhere^90^. All capture and handling procedures were based on the Canadian Council on Animal Care and the American Society of Mammologists guidelines and were approved annually by the University of Saskatchewan’s Committee on Animal Care and Supply and by the Alberta Environment and Parks Animal Care Committee (Animal use Protocol Number 20010016). This study was carried out in compliance with the Animal Research: Reporting of In Vivo Experiments (ARRIVE) guidelines.

### Sample collection and processing

All procedures for sample collection, protein extraction, and protein digestion were followed as previously described^28^. Briefly, a single skin biopsy was collected from the outer thigh of a grizzly bear using a 4–6 mm biopsy punch during April to October. Samples were collected from 95 individuals, 8 of which had more than one sample collected (103 samples total), and across 7 years (2013–2019); however, a total of 86 samples from 86 individuals were used for the mixed model analysis (see Supplementary Table S2 online). Proteins were digested using trypsin (5 µL at 1 mg/mL; Worthington Biochemical Corporation, Lakewood, NJ, USA) at a 20:1 substrate:enzyme ratio. A mix of light peptides was used to prepare the standard curve and stable isotope-labeled standard (SIS) peptides were used as internal standards for the individual and pooled samples, the standard curve samples, and the curve quality control (QC) samples. All protein extraction and digestion methods as well as the liquid chromatography and multiple reaction monitoring mass spectrometry (LC-MRM/MS) methods were completed at The University of Victoria Genome BC Proteomics Centre (Victoria, BC, Canada).

### Liquid chromatography and multiple reaction monitoring mass spectrometry assay (LC-MRM/MS) for targeted analysis

A panel of 19 proteins with established relationships to physiological function were quantitated by peptide-based analysis using LC-MRM/MS (see Wilson et al., 2020 for details on all 19 proteins). Details regarding the development and validation of the peptide panel as well as the LC-MRM/MS targeted assay parameters are provided elsewhere^28^. In short, tryptic digests were analyzed using a LC coupled with a triple quadrupole mass spectrometer (Agilent 1290 and 6495, respectively; Agilent Technologies, Santa Clara, CA, USA). Peptides were empirically optimized by analysis of the purified SIS peptides using Skyline-daily Quantitative Analysis software (version 19.1.1.248, MacCoss Laboratory, University of Washington, Seattle, WA, USA). Pooled samples were analyzed for sample preparation and MS analysis consistency and the standard curve was used to calculate the peptide concentration in fmol/injection of skin protein in samples through linear regression using the light to SIS peak area ratios (< 20% deviation in the curve QC’s level’s accuracy)^24,25^. Proteins were represented by the peptide with the greatest average concentration across all samples.

### Potential drivers of protein expression

Procedures for carrying out landscape extractions were followed as previously described^63^. Briefly, we generated both dynamic (annually varying) and static geo-spatial variables from remotely sensed data for 2013–2019. Candidate variables represented terrain conditions (static), as well as anthropogenic disturbance and food resources (dynamic), all of which have been shown to influence survival, behavior, and/or physiological function in bears^52–55^. Candidate variables were extracted and summarized at the GPS-point scale for each individual, year, and season group to account for individual and temporal variation, resulting in either a mean value for the corresponding GPS points or the proportion of GPS points within a feature, using both discrete vector and continuous raster datasets (see Supplementary Table S3 online). The majority of samples (95/103) were collected from bears that only have 1 year of corresponding landscape data. Since GPS collars were fitted at the time of sampling, it was assumed that the landscape data collected throughout the home range of an individual following the sampling event was similar to the home range when the sample was collected^92,93^ (see Supplementary Figure S2 online). Furthermore, we did not know the landscape condition of bears that were translocated, as GPS collars were fitted at the time of transport and sample collection; therefore, these individuals were excluded from the overall analysis. The exact timeframe that protein expression in grizzly bear skin represents is unknown and thus it was assumed that individual, year, and seasonal combination landscape data would account for any individual and temporal variation as well as exposures to disturbance within a reasonable timeframe (see Supplementary Table S2 online for ranges of day in year). All extractions were completed using ESRI’s ArcMap 10.3 GIS software.

Anthropogenic disturbance was represented by vector data for locations of roads, railway and power lines, oil and gas well sites, coal mines, forest harvesting (cutblocks), and protected areas. These features were mapped by the FRIGBP using data from the Human Footprint Inventory (HFI) developed by the Alberta Biodiversity Monitoring Institute (ABMI). We calculated the mean GPS points with a distance to feature value for all anthropogenic disturbance features. The proportion of GPS points per bear, year, and season group that fell within a protected area was used to represent protected area.

Food resources included variables that represent forest conditions and were used as surrogates for the distribution and abundance of foods^52,94^. We used raster data originally derived from Landsat 5 and 7 ETM imagery and a digital elevation model (DEM) to represent crown closure, percent conifer, and land cover (upland and wetland herbaceous areas)^91^. Mean GPS points with a percent conifer and crown closure value for each bear, year, and season group were calculated to represent forest cover and the number of conifers present. Upland and wetland herbaceous areas were defined as binary variables (0 = no upland/wetland herbaceous area, 1 = upland/wetland herbaceous area) and the proportion of GPS points per bear, year, and season that fell within these areas was calculated to represent areas with food resources (shrubs) near water.

Terrain conditions were represented by elevation, compound topographic index (CTI), terrain ruggedness index (TRI), slope, alpine boundary, and mean daily movement rate. Soil moisture based on slope and upstream water sources were represented by CTI^95^, while the complexity and variability of the terrain was measured by TRI^96,97^. The mean GPS points with a corresponding terrain value was calculated for each bear, year, and season group. The natural alpine region was defined as ≥ 1692 m and generated as an alpine boundary, where the proportion of GPS points per bear, year, season group that fell within this boundary was calculated. Mean daily total distance was generated for each bear, year, and season group by calculating the distance a bear traveled between GPS points.

Biological characteristics (reproductive and age class) were determined by observation at the time of capture and handling. Age class was confirmed by counting the cementum annuli of an extracted premolar from each individual^98,99^. Reproductive and age class were included as a model because previous studies have shown that home ranges size vary between males and females, and adults and subadults^64^; these classes also respond differently to features on the landscape^52,100,101^.

### Statistical analyses

To test the hypothesis that the mean expression of target proteins related to energetics, reproduction, and stress was associated with landscape condition, we used an information theoretic approach to compare 11 generalized linear mixed models (GLMMs) to identify a minimum adequate model from the set of models associated with specific hypotheses. These a priori models described plausible relationships between protein expression and landscape variables based on the literature (Table 2)^52–55^. For data exploration, we first used Cleveland dot-plots to evaluate potential outliers for the expression of each protein and removed one outlier for each of the following proteins: adiponectin, apolipoprotein, transthyretin, and corticosteroid-binding globulin^102^. We used pair-plots (Pearson r ≥ 0.70) to identify collinear proteins and landscape variables (see Supplementary Tables S4 and S5 online)^102–104^. In an attempt to not measure the same signal unnecessarily, highly correlated proteins were removed so that at least one representative of each category was present as well as those that may be suitable biomarkers based on previous grizzly bear studies and according to specific management questions. Covariates were removed if they were highly correlated with multiple other variables, so that each covariate in the models could have a unique and appropriate hypothesis. Variance inflation factors (VIFs) were then calculated for each covariate and those with a VIF > 3.0 were removed^102^. Elevation was the only additional covariate removed (see Supplementary Table S6 online). All continuous predictor variables were standardized by subtracting the mean from the observed values and dividing by the standard deviation^104^. The relationships between response (mean protein expression in fmol of protein/µg of sample, which is always positive) and independent (landscape) variables were modelled using a γ distribution and log link^105^. Given that our study area (Alberta, Canada) has an elevation gradient from west to east, with higher elevation mountain areas in the west and lower elevation areas in the foothills to the east, we assumed grizzly bears sampled within watershed units at similar elevations would reflect similar landscape condition, regardless of latitude. Therefore, we grouped individual grizzly bears by watershed unit and mean elevation to account for any spatial dependency from samples collected in similar landscape conditions (see Supplementary Figure S3 online). Determined by the provincial government, watershed units reflect the average size of a female home range (500 km^2^) and have been used in previous monitoring studies and management efforts for this population^6^. We compared a priori models by Akaike Information Criteria corrected for small sample sizes (AICc) weights, and averaged the minimum adequate models with ΔAICc < 2 for the expression of each protein^106^. Given that the same variable was not in multiple models, we used the conditional average coefficients^107^. Coefficient estimates, standard errors, and 95% confidence intervals were calculated for each protein and variables were considered significant when the 95% confidence interval did not include zero. We then predicted the probability of protein expression using the explanatory variables identified as most significant within the models. We used a paired Wilcoxon-signed rank test to investigate differences in protein expression between two time points for individuals that had more than one sample collected and an unpaired Wilcoxon-signed rank test to determine differences in protein expression between translocated and resident individuals with α = 0.05. Data were analyzed using base R and the package *glmmTMB*^108^ in R statistical software version 4.0.3 and R studio version 1.3.1093 (https://www.R-project.org/)^109^.

## Supplementary Information

Supplementary Information.

## Data Availability

The datasets generated during and/or analyzed during the current study may be requested from the corresponding author.
