# Supplementary material for: Landscape condition influences energetics, reproduction, and stress biomarkers in grizzly bears
Source: Sci Rep. 2021 Jun 9;11:12124. doi: 10.1038/s41598-021-91595-4 (PMC8190091; doi:10.1038/s41598-021-91595-4)

**Supplementary Information**

Title: Landscape condition influences energetics, reproduction, and stress biomarkers in grizzly bears

Authors: Abbey E. Wilson^1*^, Dan Wismer^3^, Gordon Stenhouse^3^, Nicholas C. Coops^2^, David M. Janz^1^

Affiliations: ^1^Department of Veterinary Biomedical Sciences, University of Saskatchewan, 44 Campus Drive, Saskatoon, Saskatchewan S7N 5B3, Canada

^2^Department of Forest Resource Management, University of British Columbia, 2424 Main Mall, Vancouver, British Columbia V6T 1Z4, Canada

^3^Foothills Research Institute, Grizzly Bear Program, 1176 Switzer Drive, Hinton, Alberta T7V 1V3, Canada

*Corresponding author: Email: abbey.wilson@usask.ca; Address: Toxicology Centre, 44 Campus Drive, Saskatoon, Saskatchewan S7N 5B3, Canada; Telephone: 1-306-966-7762; Fax: 1-306-931-1664

Table S1. Comparison of sample size corrected Akaike’s information criterion (AICc), Akaike weights (*w_i_*), number of parameters (*K*), and log likelihood (LL) for models to predict protein expression in skin samples collected from grizzly bears across Alberta, Canada. Models with ΔAICc<2 are shown in bold type.

| **Category** | **Protein** | **Model** | **Description** | **K** | **LL** | **AICc** | **ΔAICc** | ***w_i_*** |
| --- | --- | --- | --- | --- | --- | --- | --- | --- |
| Energetics | Adiponectin | **M4** | **Distance to coal mines + (1\|watershed unit)** | **4** | **-238.99** | **486.49** | **0.00** | **0.81** |
|  |  | M11 | Null + (1\|watershed unit) | 3 | -243.00 | 492.29 | 5.81 | 0.04 |
|  |  | M1 | Distance to roads + (1\|watershed unit) | 4 | -242.17 | 492.85 | 6.36 | 0.03 |
|  |  | M9 | Mean movement + (1\|watershed unit) | 4 | -242.67 | 493.84 | 7.36 | 0.02 |
|  |  | M7 | Crown closure + (1\|watershed unit) | 4 | -242.69 | 493.88 | 7.40 | 0.02 |
|  |  | M3 | Cutblock age + (1\|watershed unit) | 4 | -242.82 | 494.14 | 7.65 | 0.02 |
|  |  | M5 | Protected area + (1\|watershed unit) | 4 | -242.93 | 494.36 | 7.87 | 0.02 |
|  |  | M6 | Percent conifer + (1\|watershed unit) | 4 | -243.00 | 494.49 | 8.00 | 0.02 |
|  |  | M2 | Distance to rail/power lines + (1\|watershed unit) | 4 | -243.00 | 494.50 | 8.01 | 0.02 |
|  |  | M8 | Upland herbaceous resources + Wetland herbaceous resources + (1\|watershed unit) | 5 | -242.11 | 494.99 | 8.50 | 0.01 |
|  |  | M10 | Reproductive class + Age class + (1\|watershed unit) | 6 | -242.43 | 497.93 | 11.44 | 0.00 |
|  | Apolipoprotein | **M6** | **Percent conifer + (1\|watershed unit)** | **4** | **-137.30** | **283.09** | **0.00** | **0.33** |
|  |  | **M11** | **Null + (1\|watershed unit)** | **3** | **-139.28** | **284.85** | **1.75** | **0.14** |
|  |  | **M8** | **Upland herbaceous resources + Wetland herbaceous resources + (1\|watershed unit)** | **5** | **-137.05** | **284.86** | **1.77** | **0.14** |
|  |  | M7 | Crown closure + (1\|watershed unit) | 4 | -138.90 | 286.31 | 3.22 | 0.07 |
|  |  | M3 | Cutblock age + (1\|watershed unit) | 4 | -138.95 | 286.39 | 3.30 | 0.06 |
|  |  | M1 | Distance to roads + (1\|watershed unit) | 4 | -139.04 | 286.59 | 3.49 | 0.06 |
|  |  | M5 | Protected area + (1\|watershed unit) | 4 | -139.05 | 286.59 | 3.50 | 0.06 |
|  |  | M4 | Distance to coal mines + (1\|watershed unit) | 4 | -139.25 | 287.01 | 3.91 | 0.05 |
|  |  | M9 | Mean movement + (1\|watershed unit) | 4 | -139.26 | 287.02 | 3.92 | 0.05 |
|  |  | M2 | Distance to rail/power lines + (1\|watershed unit) | 4 | -139.27 | 287.05 | 3.96 | 0.05 |
|  |  | M10 | Reproductive class + Age class + (1\|watershed unit) | 6 | -137.67 | 288.42 | 5.33 | 0.02 |
|  | Alpha-1-acid glycoprotein | **M7** | **Crown closure + (1\|watershed unit)** | **4** | **-343.75** | **695.99** | **0.00** | **0.31** |
|  |  | **M6** | **Percent conifer + (1\|watershed unit)** | **4** | **-344.52** | **697.53** | **1.54** | **0.14** |
|  |  | **M8** | **Upland herbaceous resources + Wetland herbaceous resources + (1\|watershed unit)** | **5** | **-343.62** | **697.99** | **2.00** | **0.11** |
|  |  | M11 | Null + (1\|watershed unit) | 3 | -345.86 | 698.01 | 2.03 | 0.11 |
|  |  | M1 | Distance to roads + (1\|watershed unit) | 4 | -345.37 | 699.23 | 3.24 | 0.06 |
|  |  | M4 | Distance to coal mines + (1\|watershed unit) | 4 | -345.39 | 699.28 | 3.29 | 0.06 |
|  |  | M3 | Cutblock age + (1\|watershed unit) | 4 | -345.48 | 699.46 | 3.47 | 0.06 |
|  |  | M2 | Distance to rail/power lines + (1\|watershed unit) | 4 | -345.64 | 699.77 | 3.78 | 0.05 |
|  |  | M9 | Mean movement + (1\|watershed unit) | 4 | -345.66 | 699.82 | 3.84 | 0.05 |
|  |  | M5 | Protected area + (1\|watershed unit) | 4 | -345.85 | 700.20 | 4.22 | 0.04 |
|  |  | M10 | Reproductive class + Age class + (1\|watershed unit) | 6 | -344.67 | 702.40 | 6.42 | 0.01 |
|  | Transthyretin | **M11** | **Null + (1\|watershed unit)** | **3** | **-343.42** | **693.13** | **0.00** | **0.19** |
|  |  | **M4** | **Distance to coal mines + (1\|watershed unit)** | **4** | **-342.33** | **693.15** | **0.02** | **0.19** |
|  |  | **M5** | **Protected area + (1\|watershed unit)** | **4** | **-342.95** | **694.40** | **1.27** | **0.10** |
|  |  | **M2** | **Distance to rail/power lines + (1\|watershed unit)** | **4** | **-342.99** | **694.48** | **1.35** | **0.10** |
|  |  | **M7** | **Crown closure + (1\|watershed unit)** | **4** | **-343.05** | **694.59** | **1.46** | **0.09** |
|  |  | **M9** | **Mean daily movement + (1\|watershed unit)** | **4** | **-343.08** | **694.66** | **1.53** | **0.09** |
|  |  | **M3** | **Cutblock age + (1\|watershed unit)** | **4** | **-343.14** | **694.79** | **1.66** | **0.08** |
|  |  | M6 | Percent conifer + (1\|watershed unit) | 4 | -343.39 | 695.28 | 2.15 | 0.07 |
|  |  | M1 | Distance to roads + (1\|watershed unit) | 4 | -343.41 | 695.33 | 2.20 | 0.06 |
|  |  | M8 | Upland herbaceous resources + Wetland herbaceous resources + (1\|watershed unit) | 5 | -343.41 | 697.57 | 4.44 | 0.02 |
|  |  | M10 | Reproductive class + Age class + (1\|watershed unit) | 6 | -342.99 | 699.05 | 5.92 | 0.01 |
| Reproduction | Ceruloplasmin | **M10** | **Reproductive class + Age class + (1\|watershed unit)** | **6** | **-195.30** | **403.66** | **0.00** | **0.34** |
|  |  | **M8** | **Upland herbaceous resources + Wetland herbaceous resources + (1\|watershed unit)** | **5** | **-196.52** | **403.80** | **0.14** | **0.32** |
|  |  | **M9** | **Mean daily movement + (1\|watershed unit)** | **4** | **-198.19** | **404.88** | **1.22** | **0.18** |
|  |  | M5 | Protected area + (1\|watershed unit) | 4 | -199.42 | 407.32 | 3.67 | 0.05 |
|  |  | M2 | Distance to rail/power lines + (1\|watershed unit) | 4 | -200.15 | 408.80 | 5.14 | 0.03 |
|  |  | M3 | Cutblock age + (1\|watershed unit) | 4 | -200.21 | 408.91 | 5.25 | 0.03 |
|  |  | M6 | Percent conifer + (1\|watershed unit) | 4 | -200.61 | 409.71 | 6.05 | 0.02 |
|  |  | M11 | Null + (1\|watershed unit) | 3 | -201.85 | 410.00 | 6.34 | 0.01 |
|  |  | M4 | Distance to coal mines + (1\|watershed unit) | 4 | -201.14 | 410.77 | 7.12 | 0.01 |
|  |  | M1 | Distance to roads + (1\|watershed unit) | 4 | -201.33 | 411.15 | 7.49 | 0.01 |
|  |  | M7 | Crown closure + (1\|watershed unit) | 4 | -201.54 | 411.57 | 7.91 | 0.01 |
|  | Fetuin-B | **M7** | **Crown closure + (1\|watershed unit)** | **4** | **-262.33** | **533.16** | **0.00** | **0.23** |
|  |  | **M9** | **Mean daily movement + (1\|watershed unit)** | **4** | **-262.58** | **533.65** | **0.49** | **0.18** |
|  |  | **M5** | **Protected area + (1\|watershed unit)** | **4** | **-262.84** | **534.17** | **1.01** | **0.14** |
|  |  | **M8** | **Upland herbaceous resources + Wetland herbaceous resources + (1\|watershed unit)** | **5** | **-261.95** | **534.65** | **1.50** | **0.11** |
|  |  | **M11** | **Null + (1\|watershed unit)** | **3** | **-264.21** | **534.71** | **1.55** | **0.11** |
|  |  | M2 | Distance to rail/power lines + (1\|watershed unit) | 4 | -263.54 | 535.58 | 2.43 | 0.07 |
|  |  | M6 | Percent conifer + (1\|watershed unit) | 4 | -263.76 | 536.01 | 2.85 | 0.06 |
|  |  | M1 | Distance to roads + (1\|watershed unit) | 4 | -264.15 | 536.79 | 3.63 | 0.04 |
|  |  | M4 | Distance to coal mines + (1\|watershed unit) | 4 | -264.18 | 536.85 | 3.69 | 0.04 |
|  |  | M3 | Cutblock age + (1\|watershed unit) | 4 | -264.20 | 536.90 | 3.75 | 0.04 |
|  |  | M10 | Reproductive class + Age class + (1\|watershed unit) | 6 | -263.54 | 540.15 | 6.99 | 0.01 |
|  | Serpin B5 | **M8** | **Upland herbaceous resources + Wetland herbaceous resources + (1\|watershed unit)** | **5** | **-291.26** | **593.26** | **0.00** | **0.50** |
|  |  | **M1** | **Distance to roads + (1\|watershed unit)** | **4** | **-293.14** | **594.77** | **1.50** | **0.24** |
|  |  | M11 | Null + (1\|watershed unit) | 3 | -295.54 | 597.37 | 4.11 | 0.07 |
|  |  | M2 | Distance to rail/power lines + (1\|watershed unit) | 4 | -295.02 | 598.53 | 5.27 | 0.04 |
|  |  | M5 | Protected area + (1\|watershed unit) | 4 | -295.18 | 598.85 | 5.59 | 0.03 |
|  |  | M6 | Percent conifer + (1\|watershed unit) | 4 | -295.27 | 599.04 | 5.78 | 0.03 |
|  |  | M4 | Distance to coal mines + (1\|watershed unit) | 4 | -295.32 | 599.13 | 5.86 | 0.03 |
|  |  | M7 | Crown closure + (1\|watershed unit) | 4 | -295.40 | 599.30 | 6.04 | 0.03 |
|  |  | M3 | Cutblock age + (1\|watershed unit) | 4 | -295.53 | 599.56 | 6.30 | 0.02 |
|  |  | M9 | Mean daily movement + (1\|watershed unit) | 4 | -295.54 | 599.57 | 6.30 | 0.02 |
|  |  | M10 | Reproductive class + Age class + (1\|watershed unit) | 6 | -294.95 | 602.97 | 9.71 | 0.00 |
| Stress | Complement C3 | **M6** | **Percent conifer + (1\|watershed unit)** | **4** | **-293.56** | **595.62** | **0.00** | **0.89** |
|  |  | M8 | Upland herbaceous resources + Wetland herbaceous resources + (1\|watershed unit) | 5 | -294.87 | 600.50 | 4.88 | 0.08 |
|  |  | M11 | Null + (1\|watershed unit) | 3 | -299.34 | 604.96 | 9.35 | 0.01 |
|  |  | M5 | Protected area + (1\|watershed unit) | 4 | -298.86 | 606.21 | 10.59 | 0.00 |
|  |  | M2 | Distance to rail/power lines + (1\|watershed unit) | 4 | -298.89 | 606.27 | 10.66 | 0.00 |
|  |  | M7 | Crown closure + (1\|watershed unit) | 4 | -298.89 | 606.27 | 10.66 | 0.00 |
|  |  | M9 | Mean daily movement + (1\|watershed unit) | 4 | -299.08 | 606.66 | 11.04 | 0.00 |
|  |  | M4 | Distance to coal mines + (1\|watershed unit) | 4 | -299.18 | 606.85 | 11.23 | 0.00 |
|  |  | M1 | Distance to roads + (1\|watershed unit) | 4 | -299.24 | 606.98 | 11.36 | 0.00 |
|  |  | M3 | Cutblock age + (1\|watershed unit) | 4 | -299.33 | 607.16 | 11.54 | 0.00 |
|  |  | M10 | Reproductive class + Age class + (1\|watershed unit) | 6 | -297.22 | 607.51 | 11.89 | 0.00 |
|  | Corticosteroid-binding globulin | **M6** | **Percent conifer + (1\|watershed unit)** | **4** | **-106.18** | **220.85** | **0.00** | **0.30** |
|  |  | **M11** | **Null + (1\|watershed unit)** | **3** | **-107.98** | **222.25** | **1.40** | **0.15** |
|  |  | **M7** | **Crown closure + (1\|watershed unit)** | **4** | **-106.93** | **222.37** | **1.51** | **0.14** |
|  |  | M2 | Distance to rail/power lines + (1\|watershed unit) | 4 | -107.44 | 223.38 | 2.52 | 0.08 |
|  |  | M5 | Protected area + (1\|watershed unit) | 4 | -107.49 | 223.48 | 2.63 | 0.08 |
|  |  | M9 | Mean daily movement + (1\|watershed unit) | 4 | -107.65 | 223.80 | 2.95 | 0.07 |
|  |  | M1 | Distance to roads + (1\|watershed unit) | 4 | -107.84 | 224.18 | 3.33 | 0.06 |
|  |  | M4 | Distance to coal mines + (1\|watershed unit) | 4 | -107.89 | 224.28 | 3.43 | 0.05 |
|  |  | M3 | Cutblock age + (1\|watershed unit) | 4 | -107.97 | 224.45 | 3.59 | 0.05 |
|  |  | M8 | Upland herbaceous resources + Wetland herbaceous resources + (1\|watershed unit) | 5 | -107.64 | 226.05 | 5.19 | 0.02 |
|  |  | M10 | Reproductive class + Age class + (1\|watershed unit) | 6 | -107.93 | 228.94 | 8.09 | 0.01 |
|  | Superoxide dismutase | **M11** | **Null + (1\|watershed unit)** | **3** | **-353.94** | **714.16** | **0.00** | **0.18** |
|  |  | **M2** | **Distance to rail/power lines + (1\|watershed unit)** | **4** | **-352.84** | **714.18** | **0.02** | **0.18** |
|  |  | **M6** | **Percent conifer + (1\|watershed unit)** | **4** | **-353.37** | **715.24** | **1.08** | **0.11** |
|  |  | **M7** | **Crown closure + (1\|watershed unit)** | **4** | **-353.43** | **715.35** | **1.18** | **0.10** |
|  |  | **M4** | **Distance to coal mines + (1\|watershed unit)** | **4** | **-353.51** | **715.52** | **1.35** | **0.09** |
|  |  | **M1** | **Distance to roads + (1\|watershed unit)** | **4** | **-353.52** | **715.53** | **1.36** | **0.09** |
|  |  | **M5** | **Protected area + (1\|watershed unit)** | **4** | **-353.70** | **715.88** | **1.72** | **0.08** |
|  |  | M3 | Cutblock age + (1\|watershed unit) | 4 | -353.87 | 716.24 | 2.08 | 0.06 |
|  |  | M9 | Mean daily movement + (1\|watershed unit) | 4 | -353.93 | 716.35 | 2.19 | 0.06 |
|  |  | M8 | Upland herbaceous resources + Wetland herbaceous resources + (1\|watershed unit) | 5 | -353.43 | 717.62 | 3.45 | 0.03 |
|  |  | M10 | Reproductive class + Age class + (1\|watershed unit) | 6 | -353.33 | 719.72 | 5.55 | 0.01 |

Table S2. Overview of categorical variables used in generalized linear mixed model analysis. Landscape variables were extracted at the GPS-point level for each individual/year/season combination to account for individual and temporal variation. The watershed unit/elevation combination was used as a random effect to account for any spatial dependency between samples collected in similar landscape conditions. Reproductive class and age class were used as fixed effects in one model.

| **Variable** | **Levels** | **Number of Samples** |
| --- | --- | --- |
| Individual | 86 individuals | 86 |
| Year | 2013 | 10 |
|  | 2014 | 14 |
|  | 2015 | 21 |
|  | 2016 | 15 |
|  | 2017 | 9 |
|  | 2018 | 8 |
|  | 2019 | 9 |
| Season | Spring | 64 (Day of the year 104-162) |
|  | Summer | 5 (Day of the year 170-185) |
|  | Fall | 17 (Day of the year 220-281) |
| Geographic location (watershed unit/elevation in Alberta, Canada) | A (801-1217m) | 14 |
|  | B (1223-1281m) | 14 |
|  | C (1342-1448m) | 14 |
|  | D (1463-1548m) | 12 |
|  | E (1570-1782m) | 16 |
|  | F (1823-2261m) | 16 |
| Reproductive class | Male | 49 |
|  | Solitary Female | 27 |
|  | Female with cubs | 10 |
| Age Class | Adult (≥5 years old) | 60 |
|  | Subadult (<5 years old) | 26 |

Table S3. Calculation of landscape variables extracted at the GPS-point level for each individual/year/season combination.

| **GPS point extraction variables** | **Data calculation** |
| --- | --- |
| Distance to cutblocks | Mean GPS points with a distance to cutblock value per bear-year-season |
| Distance to oil/gas well sites | Mean GPS points with a distance to oil/gas wellsite value for each bear-year-season |
| Cutblock age | Mean GPS points with a cutblock age value for each bear-year-season |
| Distance to coal mines | Mean GPS points with a distance to coal mine value per bear-year-season |
| Distance to rail and power lines | Mean GPS points with a distance to rail and powerline value for each bear-year-season |
| Distance to roads | Mean GPS points with a distance to roads value for each bear-year-season |
| Protected area | Proportion of GPS points per bear-year-season that fell within a protected area |
| Percent conifer | Mean GPS points with a percent conifer value for each bear-year-season |
| Crown closure | Mean GPS points with a crown closure value for each bear-year-season |
| Upland herbaceous area | Proportion of GPS points per bear-year-season that fell within upland herbaceous areas (binary variable (0=no upland herbaceous area, 1=upland herbaceous area) |
| Wetland herbaceous area | Proportion of GPS points per bear-year-season that fell within wetland herbaceous areas (binary variable (0=no wetland herbaceous area, 1=wetland herbaceous area) |
| Elevation | Mean GPS points with an elevation for each bear-year-season |
| Compound topographic index (CTI) | Mean GPS points with a CTI for each bear-year-season |
| Terrain ruggedness index (TRI) | Mean GPS points with a TRI for each bear-year-season |
| Slope | Mean GPS points with a slope value per bear-year-season |
| Alpine boundary | Proportion of GPS points per bear-year-season that fell within an alpine boundary (≥1692m) |
| Daily movement | Mean daily total distance (movement) per bear-year-season |

Table S4. Highly correlated proteins (Pearson >0.7) were removed so that at least one representative of each category was present as well as those that may be suitable biomarkers based on previous grizzly bear studies and according to specific management questions. Proteins shown in bold text were selected for further analysis. The abbreviation corr refers to correlation.

| Pair | Category 1 | Protein 1 | Biomarker 1 | Category 2 | Protein 2 | Biomarker 2 | Corr |
| --- | --- | --- | --- | --- | --- | --- | --- |
| 1 | Energetics | **Adiponectin** | Metabolic disease | Stress | GRP78/BIP | Stress, pathogenesis | 0.76 |
| 2 | Energetics | **Adiponectin** | Metabolic disease | Stress | Annexin | Inflammatory disease | 0.82 |
| 3 | Energetics | **Adiponectin** | Metabolic disease | Stress | Endoplasmin | Stress, pathogenesis | 0.81 |
| 4 | Reproduction | Afamin | Pregnancy, metabolic disease | Reproduction | **Ceruloplasmin** | Pregnancy | 0.74 |
| 5 | Reproduction | Afamin | Pregnancy, metabolic disease | Reproduction | **Complement C3** | Impaired immune response | 0.78 |
| 6 | Stress | Annexin | Inflammatory disease | Stress | Endoplasmin | Stress, pathogenesis | 0.79 |
| 7 | Stress | Annexin | Inflammatory disease | Stress | **SOD** | Inflammatory and infectious disease | 0.74 |
| 8 | Stress | **CBG** | Acute and chronic inflammation | Stress | Kininogen | Inflammatory disease | 0.87 |
| 9 | Energetics | Clusterin | Degenerative diseases and tumorigenesis | Stress | Kininogen | Inflammatory disease | 0.79 |
| 10 | Reproduction | **Complement C3** | Impaired immune response | Stress | α-2-macroglobulin | Inflammatory and liver disease | 0.85 |
| 11 | Stress | GRP78/BIP | Stress, pathogenesis | Stress | Annexin | Inflammatory disease | 0.79 |
| 12 | Stress | GRP78/BIP | Stress, pathogenesis | Stress | Endoplasmin | Stress, pathogenesis | 0.86 |
| 13 | Reproduction | Prostaglandin F synthase 1 | Pregnancy, parturition | Stress | **CBG** | Acute and chronic inflammation | 0.71 |
| 14 | Energetics | **Transthyretin** | Protein-calorie malnutrition | Energetics | Vitamin D | Liver and renal disease, pregnancy | 0.74 |
| 15 | Energetics | **Transthyretin** | Protein-calorie malnutrition | Energetics | Clusterin | Degenerative diseases and tumorigenesis | 0.74 |
| 16 | Energetics | Vitamin D | Liver and renal disease, pregnancy | Reproduction | Afamin | Pregnancy, metabolic disease | 0.84 |
| 17 | Energetics | Vitamin D | Liver and renal disease, pregnancy | Reproduction | **Fetuin-B** | Female fertility | 0.72 |

Table S5. Highly correlated landscape variables (Pearson >0.7) were removed from further analysis. Landscape variables shown in bold text were selected for further analysis.

| **Variable 1** | **Variable 2** | **Correlation** |
| --- | --- | --- |
| Alpine boundary | **Elevation** | 0.78 |
| Alpine boundary | Slope | 0.74 |
| Alpine boundary | **Terrain ruggedness index** | 0.75 |
| Compound topographic index | **Elevation** | -0.70 |
| Compound topographic index | **Slope** | -0.87 |
| Compound topographic index | **Terrain ruggedness index** | -0.86 |
| Distance to cutblocks | **Protected area** | 0.87 |
| Distance to cutblocks | Distance to oil/gas wellsite | 0.85 |
| Distance to oil/gas wellsite | **Protected area** | 0.81 |
| **Distance to roads** | Distance to oil/gas wellsite | 0.73 |
| **Distance to roads** | Alpine boundary | 0.73 |
| **Elevation** | Slope | 0.82 |
| **Elevation** | Terrain ruggedness index | 0.81 |
| Slope | Terrain ruggedness index | 1.00 |

Table S6. Landscape variables with a variance inflation factor >3 were removed from further analysis.

| **Variable** | **Variance inflation factor** |
| --- | --- |
| Cutblock age | 2.24 |
| Distance to coal mines | 2.01 |
| Distance to railway and power lines | 2.53 |
| Distance to roads | 2.56 |
| Protected area | 2.59 |
| Percent conifer | 1.52 |
| Crown closure | 1.90 |
| Upland herbaceous resources | 1.32 |
| Wetland herbaceous resources | 1.21 |
| **Elevation** | **3.01** |
| Mean daily movement | 1.43 |

Figure S1. The expression of superoxide dismutase was significantly (P<0.05) elevated in resident (n=7) compared to translocated (n=7) bears. Figure was created using R statistical software version 4.0.3 and R studio version 1.3.1093 (https://www.R-project.org/).


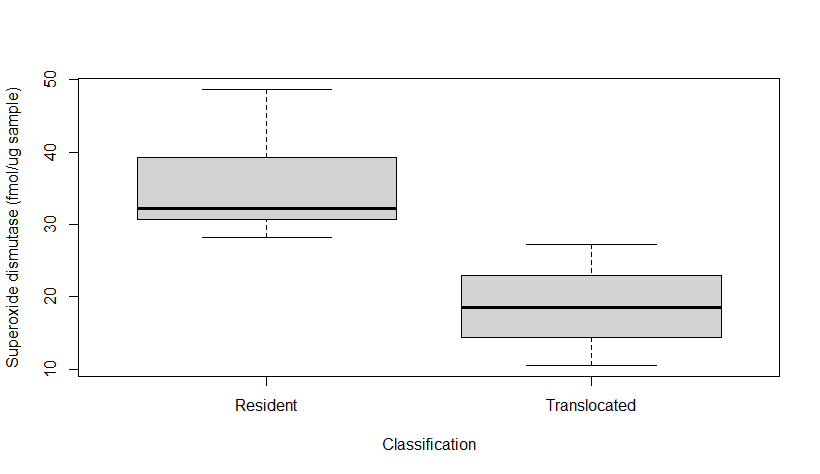


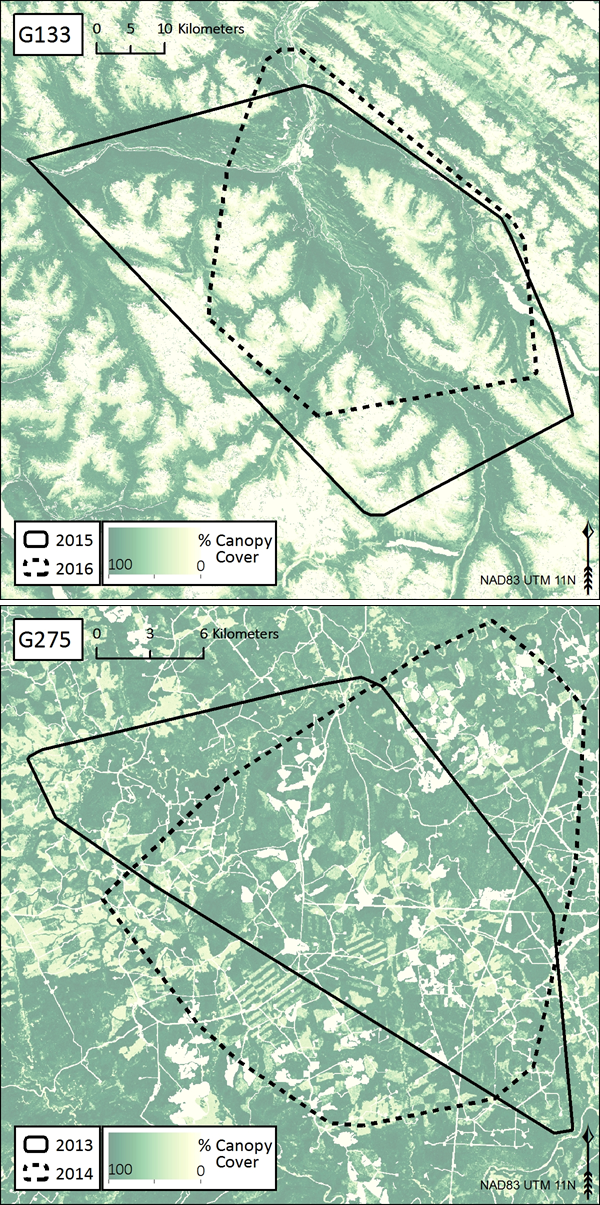
Figure S2. Home ranges of an adult male (G133) and a solitary adult female (G275) grizzly bear across two years in Alberta, Canada. Using ArcGIS Desktop 10.3.1 (https://desktop.arcgis.com/en/), a select by attribute query was used to subset GPS points by name and year. For each bear name and year group, the minimum convex polygon was generated to create home range boundaries. Canopy cover (https://developers.google.com/earth-engine/datasets/catalog/NASA_MEASURES_GFCC_TC_v3), updated using ABMI HFI18 (https://abmi.ca/home/data-analytics/da-top/da-product-overview/Human-Footprint-Products/HF-inventory.html) features was added.

Figure S3. Individual grizzly bears were grouped by watershed unit and mean elevation to account for any spatial dependency from samples collected in similar landscape conditions. Using ArcGIS Desktop 10.3.1 (https://desktop.arcgis.com/en/) software, provincial grizzly bear watershed units and grizzly bear management units were mapped. Elevation data obtained from the Shuttle Radar Topography Mission (https://www.usgs.gov/centers/eros/science/usgs-eros-archive-digital-elevation-shuttle-radar-topography-mission-srtm-1-arc?qt-science_center_objects=0#qt-science_center_objects) (1 arc second) was classified into 8 classes using natural breaks method.


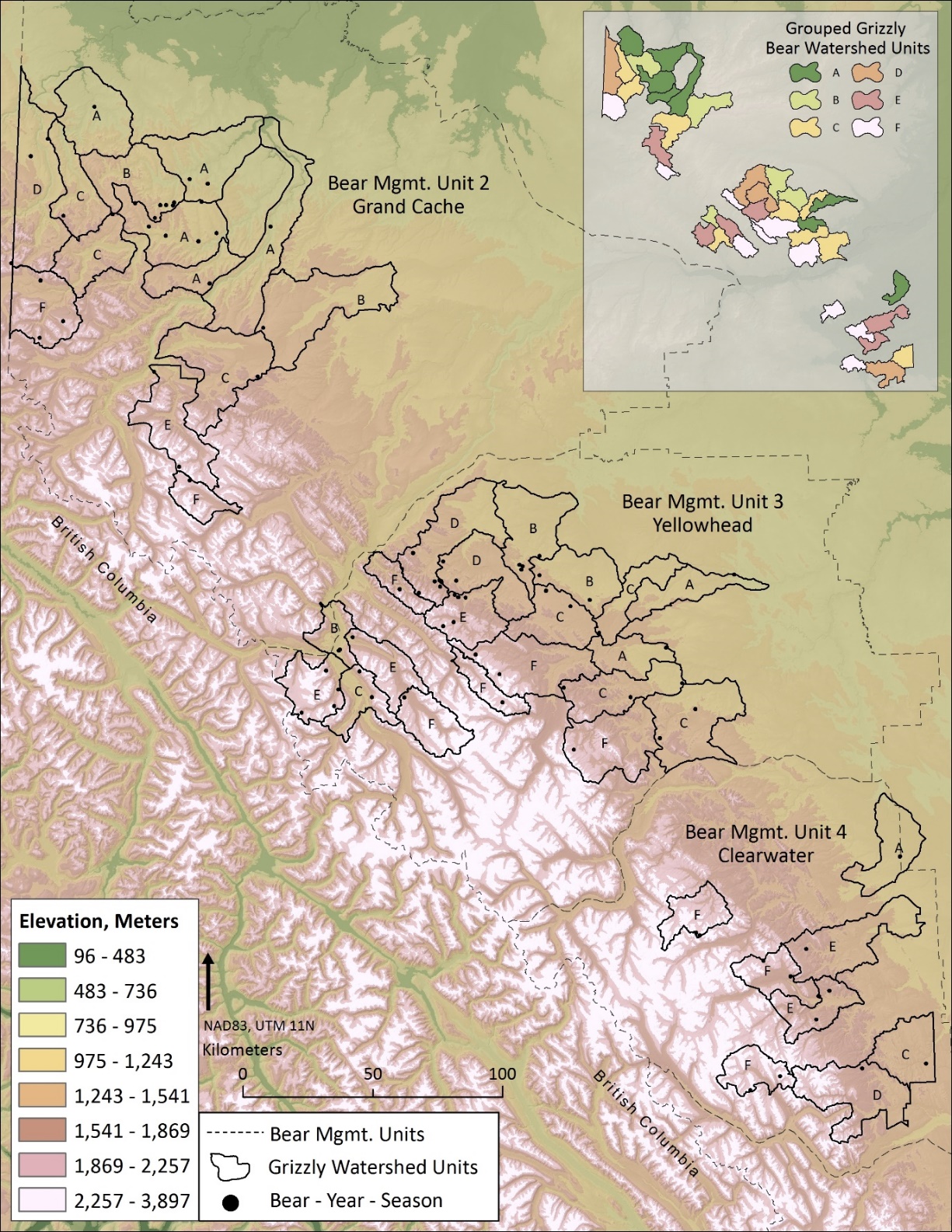

Supplement: Supplementary file 1 — Supplementary Information. [file 41598_2021_91595_MOESM1_ESM.docx]
